# Serum MMP-8: A Novel Indicator of Left Ventricular Remodeling and Cardiac Outcome in Patients after Acute Myocardial Infarction

**DOI:** 10.1371/journal.pone.0071280

**Published:** 2013-08-14

**Authors:** Marie Fertin, Gilles Lemesle, Annie Turkieh, Olivia Beseme, Maggy Chwastyniak, Philippe Amouyel, Christophe Bauters, Florence Pinet

**Affiliations:** 1 Centre Hospitalier Régional et Universitaire de Lille, Lille, France; 2 Faculté de Médecine de Lille, Lille, France; 3 Inserm, U744, Lille, France; 4 Institut Pasteur de Lille, Lille, France; 5 University Lille Nord de France, Lille, France; Penn State College of Medicine, United States of America

## Abstract

**Objective:**

Left ventricular (LV) remodeling following myocardial infarction (MI) is characterized by progressive alterations of structure and function, named LV remodeling. Although several risk factors such as infarct size have been identified, LV remodeling remains difficult to predict in clinical practice. Changes within the extracellular matrix, involving matrix metalloproteinases (MMPs) and tissue inhibitors of metalloproteinases (TIMPs), are an integral part of left ventricular (LV) remodeling after myocardial infarction (MI). We investigated the temporal profile of circulating MMPs and TIMPs and their relations with LV remodeling at 1 year and clinical outcome at 3 years in post-MI patients.

**Methods:**

This prospective multicentre study included 246 patients with a first anterior MI. Serial echocardiographic studies were performed at hospital discharge, 3 months, and 1 year after MI, and analysed at a core laboratory. LV remodeling was defined as the percent change in LV end-diastolic volume (EDV) from baseline to 1 year. Serum samples were obtained at hospital discharge, 1, 3, and 12 months. Multiplex technology was used for analysis of MMP-1, -2, -3, -8, -9, -13, and TIMP-1, -2, -3, -4 serum levels.

**Results:**

Baseline levels of MMP-8 and MMP-9 were positively associated with changes in LVEDV (*P* = 0.01 and 0.02, respectively). When adjusted for major baseline characteristics, MMP-8 levels remained an independent predictor LV remodeling (*P* = 0.025). By univariate analysis, there were positive relations between cardiovascular death or hospitalization for heart failure during the 3-year follow-up and the baseline levels of MMP-2 (*P* = 0.03), MMP-8 (*P* = 0.002), and MMP-9 (*P* = 0.03). By multivariate analysis, MMP-8 was the only MMP remaining significantly associated with clinical outcome (*P* = 0.02).

**Conclusion:**

Baseline serum MMP-8 is a significant predictor of LV remodeling and cardiovascular outcome after MI and may help to improve risk stratification.

## Introduction

Left ventricular (LV) remodeling after myocardial infarction (MI) is characterized by progressive LV dilatation [1] and associated with increased risk of heart failure and cardiovascular death [2]. Recent studies have shown that LV remodeling remains relatively frequent after MI, despite high acute reperfusion rates and widespread prescription of secondary prevention medications [3]–[4]. Although several variables – such as MI size – have been identified as risk factors [5], LV remodeling remains difficult to predict in clinical practice. The idea that biological markers might accurately predict clinical outcome is attractive, and interest is growing in identifying such markers to assess individual risk in many medical fields. We previously reviewed the published evidence about the association of circulating biomarkers with post-MI LV remodeling: a large number of the associations concerned biomarkers of extracellular matrix (ECM) turnover: matrix metalloproteinases (MMPs), and tissue inhibitors of metalloproteinases (TIMPs) [6]. Recently, guidelines for the development of biomarkers emphasize the importance of the link between the underlying pathophysiology and the predictive capabilities of the biomarkers chosen. Accordingly, biomarkers of ECM homeostasis were selected for this study because past experimental studies have shown that changes within the ECM are an integral part of post-MI LV remodeling [7]. In various animal MI models, increased MMP expression occurs during the development of heart failure [8], MMP inhibition attenuates LV remodeling [9], and TIMP deficiency accelerates adverse LV remodeling by promoting ECM degradation [10].

Clinical studies investigating the relations between circulating MMPs/TIMPs levels and LV remodeling have nonetheless produced discordant results. Most were relatively small, and the timing of the evaluation varied greatly. The latter point is especially important because LV remodeling is a time-dependent process that continues for 6 to 12 months after MI. Studies with a shorter follow-up therefore focus only on early remodeling rather than the entire process. In addition, previous studies with large cohorts have investigated limited number of MMPs/TIMPs. Most studies focused on MMP-2, MMP-9, and TIMP-1 [6] and very limited information is available on other MMPs/TIMPs such as MMP-8, TIMP-2, and TIMP-4 that could however be relevant biomarkers in this setting. In the present study, MMPs/TIMPs levels were determined with multiplex technology in samples from the REVE-2 study, which prospectively analysed the relations between circulating biomarkers and LV remodeling in 246 patients after a first anterior MI and included repeated echocardiographic examinations and serial blood sampling in the first year after MI.

## Methods

### Study Population

The design of the REVE-2 study has been published in detail elsewhere [4]. We enrolled 246 patients with a first anterior wall Q-wave MI between February 2006 and September 2008. Inclusion criteria were hospitalization within 24 hours after symptom onset and at least 3 akinetic LV segments in the infarct zone at the predischarge echocardiography. The Ethics Committee of the Centre Hospitalier et Universitaire de Lille approved the research protocol, and each patient provided written informed consent. The protocol required serial echographic studies at hospital discharge (day 3 to day 7) and 3 and 12 months after MI. Serial blood samples were taken at discharge (day 3 to day 7), and 1, 3, and 12 months after MI.

### Echocardiographic Studies

A standard echographic imaging protocol was used based on apical 4- and 2-chamber views as previously described [3]. All echocardiograms were analyzed at the Lille Core Echo Laboratory (Lille, France), as previously described [3]. LV end-diastolic volume (EDV), LV end-systolic volume (ESV), and LV ejection fraction (EF) were calculated with a modified Simpson’s rule.

### MMPs/TIMPs Measurements

For each patient, serum and plasma (ethylenediaminetetraacetic acid (EDTA) used as anticoagulant) were collected at the 4 times mentioned above and analysed in a core laboratory (Lille, France). Serum levels of MMP-1, -2, -3, -8, -9 and -13 were measured with a multiplex luminex kit for simultaneous quantitative detection of human MMPs, according to the manufacturer’s instructions (Human MMP Panel Fluorokine Multi Analyte Profiling (MAP) Kit, R&D Systems, Minneapolis, Minnesota). MMP-13 was not detected in our samples (detection limit at 0.71 ng/ml). Serum levels of TIMP-1, -2, -3 and -4 were measured with a multiplex TIMP immunoassay (Human TIMP Fluorokine MAP 4-plex Kit, R&D Systems). TIMP-3 was detected in only one third of our samples (detection limit at 7.75 ng/ml). Baseline MMP-9 levels were also measured in all patients’ plasma samples with the Milliplex MAP Human MMP Panel 2 (Millipore Corp, Billerica, MA). All samples were analysed with the Bio-Plex system (Bio-Rad Laboratories, Hercules, CA) following the manufacturer’s instructions. Experimental data were analysed by fitting a four-parameter logistic curve to the standard analyte curves.

### Clinical Follow-up

Clinical follow-up was performed at outpatient visits or by contacting the general practitioner or cardiologist between February 2009 and June 2011. We collected data on death, and hospitalization for heart failure (symptoms of dyspnea or edema associated with bilateral rales, elevated venous pressure, or interstitial or alveolar edema on chest x-ray, or the addition of intravenous diuretics or inotropic medications).

### Statistical Analysis

STATA 9.0 (STAT Corp., College Station, Texas) was used for the statistical analysis. Results are presented as the mean±SD, median with 25^th^ and 75^th^ percentiles, or frequency expressed as a percentage. Variables with skewed distribution were log-transformed before being used as continuous variables in statistical analyses. Continuous variables were compared with the unpaired Student’s *t*-test, or with simple linear regression as appropriate. Changes in MMPs/TIMPs over time were assessed by repeated-measures ANOVA with Scheffe’s post-hoc test. All hypotheses were two-tailed with a 0.05 type I error rate. LV remodeling was defined as the percent change in LVEDV from baseline to 1 year follow-up. We analyzed the predictive value of early (baseline and 1 month) levels of MMPs/TIMPs on LV remodeling. Independent correlates of change in LVEDV were identified by multiple linear regression. Variables with *P*<0.10 on univariate analysis were entered in the multivariable model. Colinearity was excluded by means of a correlation matrix between candidate predictors. To illustrate our findings, LVR was defined as a >20% increase in EDV between baseline and the 1-year follow-up examination, a definition used previously to indicate severe remodeling [3]–[4],[11]–[12]. Cox proportional hazards analyses were performed to determine predictors of clinical outcome. Cumulative survival was estimated using the Kaplan-Meier method, and the differences in survival curves were compared with a log rank test. To illustrate our findings, baseline levels of MMP-8 were categorized into tertiles.

## Results

### Baseline Characteristics

Baseline and follow-up data for the REVE-2 cohort have been published previously [4] and are summarized in Table 1. Briefly, most patients were men (mean age: 57±14 years). Initial reperfusion therapy was primary percutaneous coronary intervention in 128 patients and thrombolysis in 87; 31 patients did not undergo reperfusion therapy. Nearly all patients received secondary preventive treatments. The baseline LVEF was 49±8%.

**Table 1:** Baseline characteristics of the study population.

Age, years	57±14
Women	46 (19%)
Hypertension	89 (36%)
Diabetes mellitus	51 (21%)
Primary percutaneous coronary intervention	128 (52%)
Thrombolysis alone	28 (11%)
Thrombolysis and rescue percutaneous coronary intervention	59 (24%)
No reperfusion	31 (13%)
Heart failure (Killip class ≥2) during hospitalization	79 (32%)
LVEF, %	49±8
Antiplatelet therapy	246 (100%)
ß blockers	238 (97%)
Angiotensin-converting enzyme inhibitors	238 (97%)
Statins	231 (94%)

### Temporal Profile of MMPs/TIMPs

Table 2 summarizes serum levels of MMP-1, -2, -3, -8, -9, and TIMP-1, -2, -4 at baseline and 1, 3, and 12 months after MI. Overall, 3 temporal patterns were identified: MMP-1 levels remained stable throughout the study; levels of MMP-2, MMP-3, TIMP-2, and TIMP-4 were significantly lower at baseline and increased thereafter; in contrast, MMP-8, MMP-9 and TIMP-1 levels were significantly higher at baseline and decreased during the chronic phase.

**Table 2:** Serum levels of MMPs and TIMPs at baseline and during follow-up.

Timing	Baseline	1 month	3 months	1 year
N	236	230	230	227
**MMP-1**	3.9 [2.2–7.3]	4.0 [2.2–6.8]	4.3 [2.3–6.7]	3.8 [2.1–6.6]
**MMP-2**	177 [154–206]	205 [183–239]*	213 [188–240]*	212 [191–240]* †
**MMP-3**	16.7 [12.1–25.6]	18.1 [13.4–22.9]	17.4 [13.2–25.2]	19.4 [14.4–25.1]* † ‡
**MMP-8**	17.5 [9.5–32.1]	11.4 [6.3–21.1]*	12.4 [7.5–21.3]*	11.8 [5.5–19.6]*
**MMP-9**	518 [355–804]	474 [325–753]	502 [343–7161]	467 [285–672]§ ‡
**TIMP-1**	153 [127–188]	140 [119–163]*	135 [116–163]*	131 [110–153]* † ‡
**TIMP-2**	81 [71–93]	94 [82–106]*	93 [84–110]*	91 [83–107]*
**TIMP-4**	1.24 [0.97–1.63]	1.34 [1.08–1.89]§	1.36 [1.09–1.82]*	1.42 [1.08–1.88]* †

Concentrations are expressed in ng/ml. Results are presented as the median with 25^th^ and 75^th^ percentiles.

* *P*<0.0001 vs baseline;

† *P*<0.05 vs 1 month;

‡ *P*<0.05 vs 3 months;

§ *P*<0.05 vs baseline.

### Relations of MMPs/TIMPs with Patients Characteristics

We investigated the relations between baseline levels of MMPs/TIMPs and major baseline characteristics of the study population (Table 3). With the exception of MMP-1, -8, and -9, most MMPs/TIMPs increased with aging. Hypertension and diabetes were associated with increases in several MMPs/TIMPs, particularly MMP-3 and TIMP-4. Baseline levels of MMPs/TIMPs did not change according to reperfusion therapy. Finally, higher levels of MMP-8 and -9 were observed in patients who experienced heart failure during hospitalization and in patients with lower LVEF.

**Table 3:** Relations of baseline levels of MMPs/TIMPs with patients characteristics.

	Age	Women	Hypertension	Diabetes	Noreperfusion	Heart failure during hospitalization	LVEF
**MMP-1**	ns	ns	ns	ns	ns	ns	ns
**MMP-2**	*P*<0.0001 (pos.)	*P*<0.05 (pos.)	*P*<0.05 (pos.)	ns	ns	ns	ns
**MMP-3**	*P*<0.05 (pos.)	*P*<0.0001 (neg.)	*P*<0.01 (pos.)	*P*<0.05 (pos.)	ns	ns	ns
**MMP-8**	ns	ns	ns	*P*<0.05 (pos.)	ns	*P*<0.05 (pos.)	*P*<0.05 (pos.)
**MMP-9**	ns	*P*<0.05 (neg.)	ns	ns	ns	*P*<0.05 (pos.)	*P*<0.05 (pos.)
**TIMP-1**	*P*<0.05 (pos.)	ns	ns	ns	ns	ns	ns
**TIMP-2**	*P*<0.0001 (pos.)	ns	ns	*P*<0.05 (pos.)	ns	ns	ns
**TIMP-4**	*P*<0.0001 (pos.)	*P*<0.05 (pos.)	*P*<0.001 (pos.)	*P*<0.01 (pos.)	ns	ns	ns

pos. indicates a positive association of the variable with the MMP or TIMP while neg. indicates a negative association. ns, non significant.

### Relations of Early MMPs/TIMPs Levels with Subsequent LV Remodeling

During the 1-year follow-up period, 3 patients died (all from cardiovascular causes) and 1 patient underwent heart transplantation; these patients were excluded from the remodeling study. In addition, 3 patients had recurrent MIs during the follow-up period; these patients were not excluded from the remodeling study. Echocardiographic follow-up was achieved in 226 of the 242 eligible patients (93%, 1 patient was lost to follow-up and 15 patients declined the repeat echographic studies or had inadequate image quality for measurement of LV volumes). In the overall cohort, LV remodeling was documented by an increase in LVEDV (baseline 52.3±14.0 ml/m^2^, 1 year 62.3±18.4 ml/m^2^, *P*<0.0001). We first investigated the relations between early MMPs/TIMPs levels and LV remodeling as a quantitative variable defined as percent change in LVEDV from baseline to 1-year follow-up (Table 4). Baseline levels of MMP-8 and MMP-9 were significantly and positively associated with LV remodeling. By contrast, 1-month levels of MMP-8 and MMP-9 were not associated with LV remodeling. Other MMPs/TIMPs, whether assessed at baseline or at 1 month, were not associated with LV remodeling.

**Table 4:** Regression coefficients for univariable correlations between baseline and 1 month levels of MMPs/TIMPs and LV remodeling defined as changes in LVEDV from baseline to 1 year.

	Baseline	1 month
**MMP-1**	0.036	0.026
**MMP-2**	0.018	0.005
**MMP-3**	0.007	−0.034
**MMP-8**	0.168*	0.087
**MMP-9**	0.153†	0.056
**TIMP-1**	0.116	0.031
**TIMP-2**	0.066	0.032
**TIMP-4**	0.120	0.122

* *P* = 0.01;

† *P* = 0.02.

When LV remodeling was defined as a >20% increase in LVEDV from baseline to 1 year, it was observed in 87 patients (38% of population with echocardiographic follow-up). Baseline MMP-8 levels were significantly higher in the remodeling group (21.7 [13.5–31.8] ng/ml) than in the no remodeling group (15.4 [8.5–31.9] ng/ml), *P* = 0.02. Similar results were obtained for baseline levels of MMP-9 (526 [355–918] vs 510 [365–692] ng/ml, *P* = 0.05).

To take into account potential confounding factors, we performed multivariate analyses adjusting for major baseline characteristics (age, gender, body mass index, history of hypertension, diabetes mellitus, prior angina, reperfusion therapy, heart failure during hospitalization, final TIMI flow grade in the infarct-related vessel, systolic blood pressure, heart rate, creatine kinase levels, wall motion score index and LVEF). Because of a high correlation between MMP-8 and MMP-9 baseline levels (r = 0.766), we entered them in 2 different models (Table 5). The baseline levels of MMP-8 were independently associated with LV remodeling (*P* = 0.025); the other variable retained into the model was baseline LVEF. By contrast, the baseline levels of MMP-9 did not remain associated with LV remodeling when adjusted for baseline characteristics.

**Table 5:** Independent predictors of LV remodeling defined as changes in LVEDV from baseline to 1 year.

	Independent variables	Standardizedß coefficient	P value
**Model 1**	Baseline LVEFBaseline MMP-8	−0.2170.162	0.0040.025
**Model 2**	Baseline LVEFBaseline MMP-9	−0.1860.131	0.0050.07

Model 1: with MMP-8; Model 2: with MMP-9.

The type of sample, plasma or serum, affects measured concentrations of circulating MMP-9. As MMP-9 was measured in plasma in most studies, we also measured it in plasma of 246 patients of the REVE 2 study to strengthen our data. Baseline plasma MMP-9 concentration was lower than in serum with a median value of 33 [19–54] ng/ml compared to 518 [355–804] ng/ml in serum. Baseline plasma MMP-9 level was not associated with change in LVEDV from baseline to 1 year as a quantitative variable (*P* = 0.167); however, baseline plasma MMP-9 levels were higher in patients with >20% change in LVEDV (39 [19–70] ng/ml) compared to patients with <20% change in LVEDV (29 [18–49] ng/ml), *P* = 0.02).

### Clinical Endpoint and Prediction of Outcome

**Figure 1:**
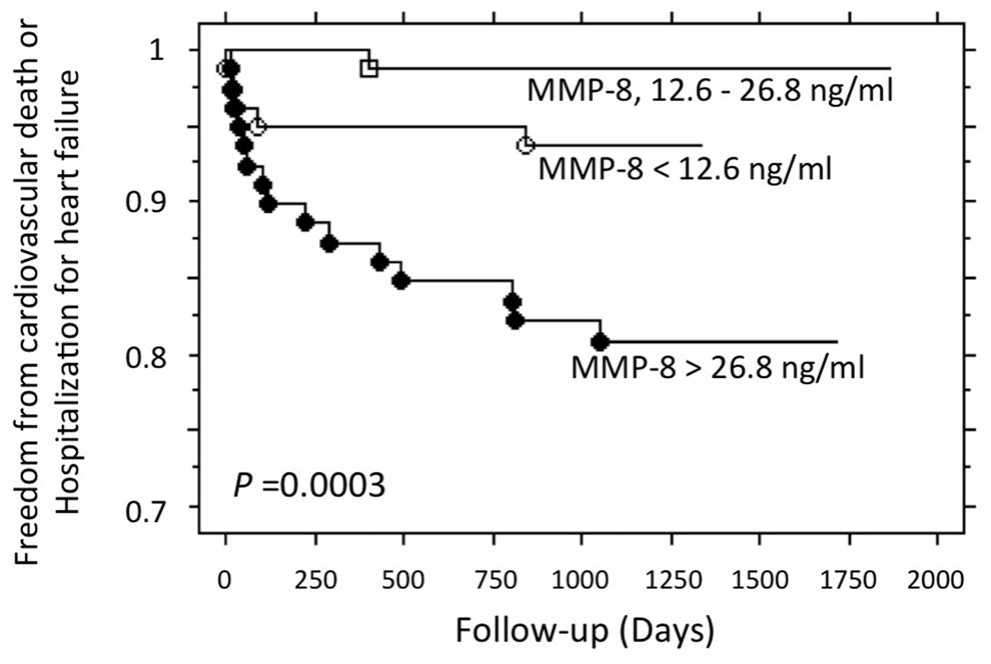
Kaplan-Meier Meier survival curves according to baseline levels of MMP-8. MMP-8 levels were categorized into tertiles: first tertile (n = 79) = MMP-8<12.6 ng/ml; second tertile (n = 78) = MMP-8 between 12.6 and 26.8 ng/ml; third tertile (n = 79) = MMP-8>26.8 ng/ml.

Clinical follow-up data were obtained for 245 patients at a mean of 1098 days; 1 patient was lost to follow-up. Sixteen patients were hospitalized for heart failure and 15 patients died (11 from cardiac causes). We investigated the associations of baseline levels of MMPs/TIMPs with cardiovascular death or hospitalization for heart failure. By univariate analysis, 3 MMPs were significantly and positively associated with clinical outcome: MMP-2 (*P* = 0.03), MMP-8 (*P* = 0.002), and MMP-9 (*P* = 0.03). However, when adjusted for major baseline characteristics (age, gender, body mass index, history of hypertension, diabetes mellitus, prior angina, reperfusion therapy, heart failure during hospitalization, final TIMI flow grade in the infarct-related vessel, systolic blood pressure, heart rate, creatine kinase levels, wall motion score index and LVEF), MMP-8 was the only MMP that remained significantly associated with clinical outcome (*P* = 0.02) while MMP-2 and MMP-9 did not reach statistical significance (*P* = 0.327 and 0.311, respectively). To illustrate our results, baseline MMP-8 levels were divided into tertiles (<12.6 ng/ml (n = 79), 12.6–26.8 ng/ml (n = 78), >26.8 ng/ml (n = 79)). As shown in Figure 1, most of the clinical events during follow-up occurred in patients with MMP-8 levels >26.8 ng/ml (*P* = 0.0003).

## Discussion

This study is the first to provide an in-depth investigation of the temporal pattern of many circulating MMPs and all TIMPs during a one-year follow-up after acute MI and their relation with LV remodeling and prognosis in a large cohort of patients. Compared to previous investigations, our study has clear strengths related to its prospective design: relatively high number of patients, very homogeneous population with a first anterior ST-elevation MI, high reperfusion rate, nearly universal use of secondary prevention treatments, serial blood sampling at pre-specified time points during one year, and prospective assessment of one-year LV remodeling with core lab analysis. Another strength is related to the availability of bead-based multi-analyte profiling technology that can multiplex many MMPs or all TIMPs in a single sample and allowed us to study a large panel of biomarkers of ECM turnover.

### Changes in Circulating MMPs/TIMPs Levels during the Post-MI Period

Numerous studies of animal models have documented selective, time-dependent modulation of cardiac MMPs and TIMPs after MI [7]. Because myocardial MMPs and TIMPs during and after MI are much more difficult to measure in humans, monitoring their levels/activity in the peripheral blood has emerged as an alternative strategy. Several studies have demonstrated that changes in these circulating levels may indeed predict changes within the myocardium [13]. These MMPs/TIMPs concentrations in the peripheral blood are at best, however, an indicator of a localized process occurring within the myocardium, and various interactions might prevent some MMPs or TIMPs from entering the circulation [7].

Our study, investigating many MMPs and TIMPs by serial blood samples during a one-year follow-up in 246 patients, provides an extensive description of the temporal pattern of these proteins after MI. Specifically, MMP-8, MMP-9 and TIMP-1 peaked early after MI and subsequently decreased during the chronic stage, while MMP-2, TIMP-2 and TIMP-4 also decreased early. These data are consistent with the findings by Webb et al. [14]. Finally, we found decreased levels of MMP-3 in the MI acute phase, as previously described by Samnegard et al. [15]. The present study, performed in a much larger population, thus reinforces the concept of a specific temporal post-MI pattern of MMPs and TIMPs release and probably reflects both a local shift in cell type activation and ECM proteolytic events.

Preanalytical conditions affect the measurements of circulating MMPs and TIMPs, including the type of sample (plasma or serum), the anticoagulant used to collect plasma, and the freeze-thaw cycles. Here, we found higher MMP-9 concentrations in serum than plasma, in agreement with previous studies [16]. This difference might be attributed to the differential release of this analyte from blood cells during platelet activation or the sampling process.

### Associations of MMPs/TIMPs Levels with LV Remodeling

This study establishes for the first time a positive association between baseline levels of MMP-8 and the extent of post-MI LV remodeling. To date, only 2 reports [14], [17] have studied this topic, with negative results possibly due to limited number of patients (n = 32 and 58, respectively). Although, there is no formal demonstration in the present study that the circulating MMP-8 derives from the heart, our data are in agreement with experimental studies suggesting that increased MMP-8 activity in the infarct area, caused by greater inflammatory cell infiltration, contributes to infarct rupture in humans [18]. For MMP-9, our study confirms in serum previous findings of positive relations between plasma MMP-9 and LV remodeling [14], [19]–[22]. These studies, however were often small, and only one included >100 patients [21]. Although our results make MMP-9 one of the biomarkers most consistently associated with LV remodeling, we observed that, contrary to MMP-8, it did not remain an independent predictor when adjusted for major characteristics and particularly for baseline LVEF as an indicator of infarct size. It must also be pointed that the association of MMP-8 and MMP-9 with LV remodeling was solely observed in baseline samples, and not in 1 month samples; this suggests that the early expression of MMP-8 and MMP-9 may indicate the onset of intramyocardial processes that will ultimately lead to LV dilatation and dysfunction.

We found no association of MMP-3 and TIMP-1 with LV remodeling. Two previous studies have reported positive associations for MMP-3 [23]–[24]. Discordant results have been published for TIMP-1: one group found that TIMP-1 concentration correlated with LV volumes and remodeling in a cohort of 404 MI patients [21] but not in a separate cohort of 100 MI patients [25]. Methodological differences between the studies might explain these discrepancies. In addition, as with all negative results, our data must be interpreted taking into account the statistical power of the study; with a 2-sided alpha error of 0.05 and 80% power, our sample size allowed to detect a 10% difference in MMP-2, a 15% difference in TIMP-1 and TIMP-2, a 25% difference in TIMP-4, a 30% difference in MMP-3, and a 35% difference in MMP-1, between patients with LV remodeling and patients without LV remodeling.

### Associations of MMPs/TIMPs Levels with Clinical Outcome after MI

Because LV remodeling is associated with increased risk of death an heart failure in post-MI [2], we investigated the associations of MMPs and TIMPs to cardiovascular outcome and found that baseline levels of MMP-8 predicted prognosis after MI. MMP-8 remained a significant predictor after adjustment for major baseline characteristics including LVEF. To date only a few studies have investigated the association of MMP-8 with cardiovascular outcome. In the study of Tuomainen et al. [26], higher levels of MMP-8 were associated with the worst cardiovascular outcome in patients with atherosclerosis, possibly because of the involvement of MMP-8 in vascular matrix remodelling and rupture of unstable plaques [27]. Our study is the first to show that serum MMP-8 is an independent predictor of outcome in post-MI patients. The significant association between MMP-8 and LV remodeling, suggests that the worst cardiovascular outcomes might be explained by increased ECM myocardial remodeling.

### Clinical Implications - Conclusions

Our data on MMPs/TIMPs reinforce current knowledge about the involvement of this system in LV remodeling and heart failure. Although validation is required in large series of patients with clinical follow-up, our study suggests that the early determination of serum MMP-8 and MMP-9 levels might help to detect patients at risk of LV remodeling. In addition, early MMP-8 is an independent indicator of prognosis in post-MI patients. Early identification of patients most susceptible to LV remodeling and poor outcome might encourage more aggressive therapy for this high-risk group. Finally, it remains to be established whether modifying these changes in circulating MMPs/TIMPs profiles in the post-MI period would improve the clinical course.
